# Cryptogenic Pyogenic Liver Abscess as a Sentinel of Occult Colorectal Cancer: Successful Conservative Management of a Rare Presentation

**DOI:** 10.7759/cureus.106605

**Published:** 2026-04-07

**Authors:** Akhil Tomy, Niladri Dutta, Anupama Nair

**Affiliations:** 1 Internal Medicine, Betsi Cadwaladr University Health Board, Rhyl, GBR; 2 Geriatrics, Betsi Cadwaladr University Health Board, Rhyl, GBR

**Keywords:** atypical liver abscess, colorectal carcinoma, conservative management, cryptogenic infection, drainage of abscess, pyogenic liver abscess, sentinel presentation

## Abstract

Pyogenic liver abscess (PLA) is an uncommon but serious infection, usually linked to biliary or intra-abdominal sources. When no clear cause is identified, the possibility of an underlying gastrointestinal malignancy, especially colorectal cancer (CRC), should always be considered. In some cases, a liver abscess may be the very first sign of an otherwise silent cancer.

We report a 68-year-old woman with a history of breast cancer, chronic obstructive pulmonary disease, and non-alcoholic fatty liver disease, who presented with sepsis and confusion. Imaging revealed multiple large, multiseptated liver abscesses. Despite their size, she was treated successfully with antibiotics alone, without drainage. A few weeks later, she was readmitted with abdominal pain, iron deficiency anemia, and *Clostridium difficile* colitis. Colonoscopy revealed an ascending colonic adenocarcinoma. She underwent right hemicolectomy, and pathology confirmed stage II colonic carcinoma. Her earlier liver abscesses were later recognized as the first sign of her underlying cancer.

This case highlights the importance of evaluating cryptogenic liver abscesses for occult CRC. It also shows that, in selected patients, even large, complex abscesses can respond well to antibiotics alone. Early multidisciplinary involvement and clinical suspicion are key.

## Introduction

Pyogenic liver abscess (PLA) is an uncommon but potentially life-threatening intra-abdominal infection, with an estimated incidence of 2-3.6 per 100,000 persons in high-income countries, though this figure is rising globally [1]. The condition most often arises from biliary tract disease, intra-abdominal infections, or hematogenous spread from the portal circulation [2,3]. Traditional management has relied on a combination of broad-spectrum antibiotics and percutaneous or surgical drainage, particularly for abscesses larger than 5 cm [4].

In recent decades, significant microbial and epidemiological shifts have been observed. While Western cohorts historically reported polymicrobial infections often related to biliary disease, there has been a marked increase in monomicrobial *Klebsiella pneumoniae* infections, a trend first widely reported in Asia and now recognized globally [5,6]. This shift is of clinical importance as *K. pneumoniae* PLA is frequently associated with cryptogenic origins and metastatic septic complications [7]. A subset of PLA, termed cryptogenic, is diagnosed when no primary hepatobiliary or intra-abdominal source can be identified [8]. Growing evidence has firmly established a strong association between cryptogenic PLA and underlying colorectal cancer (CRC).

Large population-based cohort studies from Taiwan, Korea, and other regions report a three- to seven-fold increased risk of CRC in these patients, with the highest risk observed within the first year following the PLA diagnosis [9-11]. Proposed mechanisms include bacterial translocation through tumor-disrupted colonic mucosa and subsequent portal venous seeding, creating a nidus for infection in the liver [12,13]. Consequently, a cryptogenic PLA may be the first clinical manifestation of an otherwise silent colorectal malignancy [9-11,14]. This critical association underscores the imperative for a thorough lower gastrointestinal evaluation, including colonoscopy, in all patients with a cryptogenic PLA, particularly those with red flags such as unexplained iron deficiency anemia or abnormal colonic findings on imaging [14,15].

We present a diagnostically challenging case of multifocal, large, multiseptated PLA that was successfully managed with antibiotic therapy alone, in whom subsequent evaluation revealed an ascending colonic adenocarcinoma. This case underscores two key clinical considerations: the role of cryptogenic PLA as a sentinel indicator of underlying CRC, and the potential for conservative management in carefully selected patients with even large PLA, challenging conventional guidelines [16,17].

## Case presentation

A 68-year-old Caucasian female patient with a history of Grade 3 invasive ductal carcinoma (Estrogen receptor/progesterone receptor (ER/PR)-positive, post-mastectomy with adjuvant therapy), chronic obstructive pulmonary disease, and non-alcoholic fatty liver disease was found unresponsive at home. On arrival, she was febrile (40.3°C; reference range 36.1°C to 38.0°C), tachycardic (120 bpm; reference range 60 to 100 bpm), tachypneic (40 breaths/min; reference range 12 to 20 breaths/minute), and hypoxic with oxygen saturation of 85% (reference range 94-100%) on room air. Examination revealed abdominal tenderness and a strong urinary odor. Laboratory tests showed leukocytosis (18.9 × 10^9^/L; reference range: 4-11 × 10^9^/L) and markedly elevated C-reactive protein (384 mg/L; reference </=5 mg/L).

**Figure 1:**
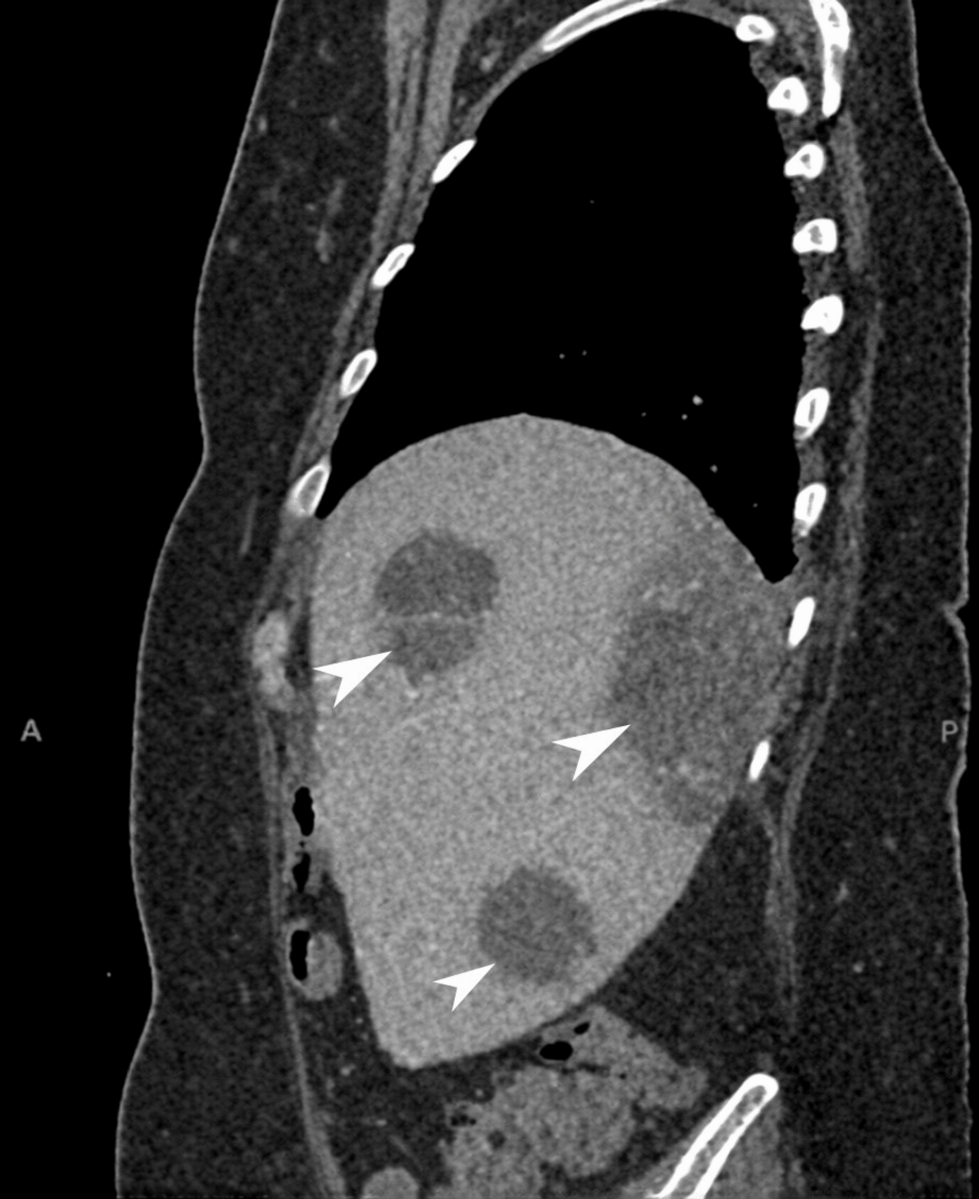
Axial contrast-enhanced CT Shows multiple low-attenuation lesions in the right hepatic lobe with peripheral rim enhancement (white arrowheads), consistent with hepatic abscesses; CT: Computerized tomography.

**Figure 2:**
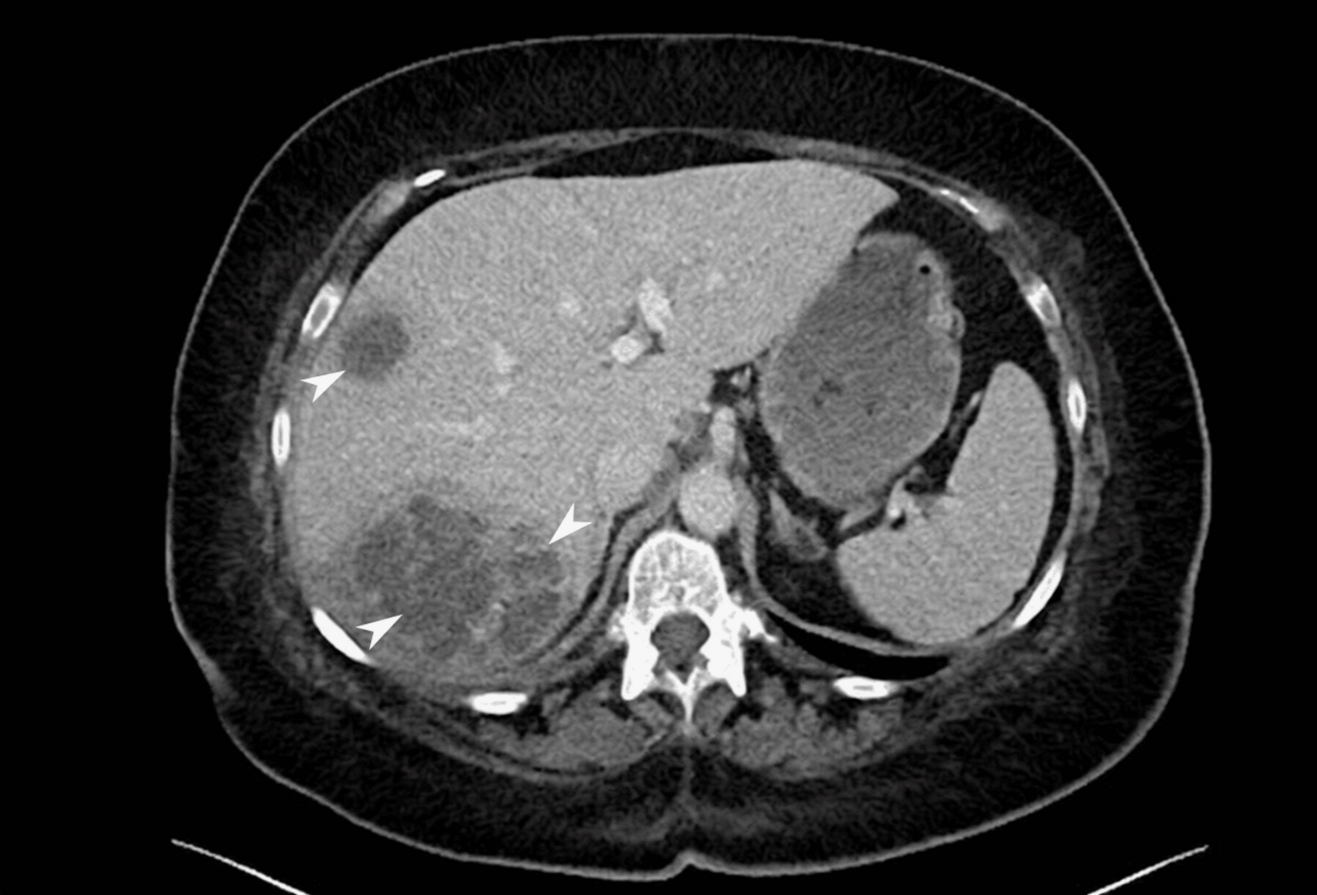
Sagittal CT image Reveals multiple rounded, low-attenuation lesions with rim enhancement (white arrowheads), supporting multifocal hepatic abscess formation; CT: Computerized tomography.

A provisional diagnosis of urosepsis was made, and empirical intravenous piperacillin-tazobactam was initiated. No growth was observed in the blood or urine cultures done at this point. Despite treatment, pyrexia and raised inflammatory markers persisted initially. By day five, CRP was 170 mg/L and white cell count 16.1 × 10^9^/L, with procalcitonin 7.1 µg/L (reference </=0.5 µg/L). Computerized tomography (CT) imaging revealed multiple multiseptated hepatic abscesses and colonic diverticulosis. Hydatid cyst and metastatic disease were excluded based on the absence of relevant travel history, regular deworming of a household dog, normal tumor markers, and no evidence of recurrent breast cancer (Figures 1, 2).

**Figure 3:**
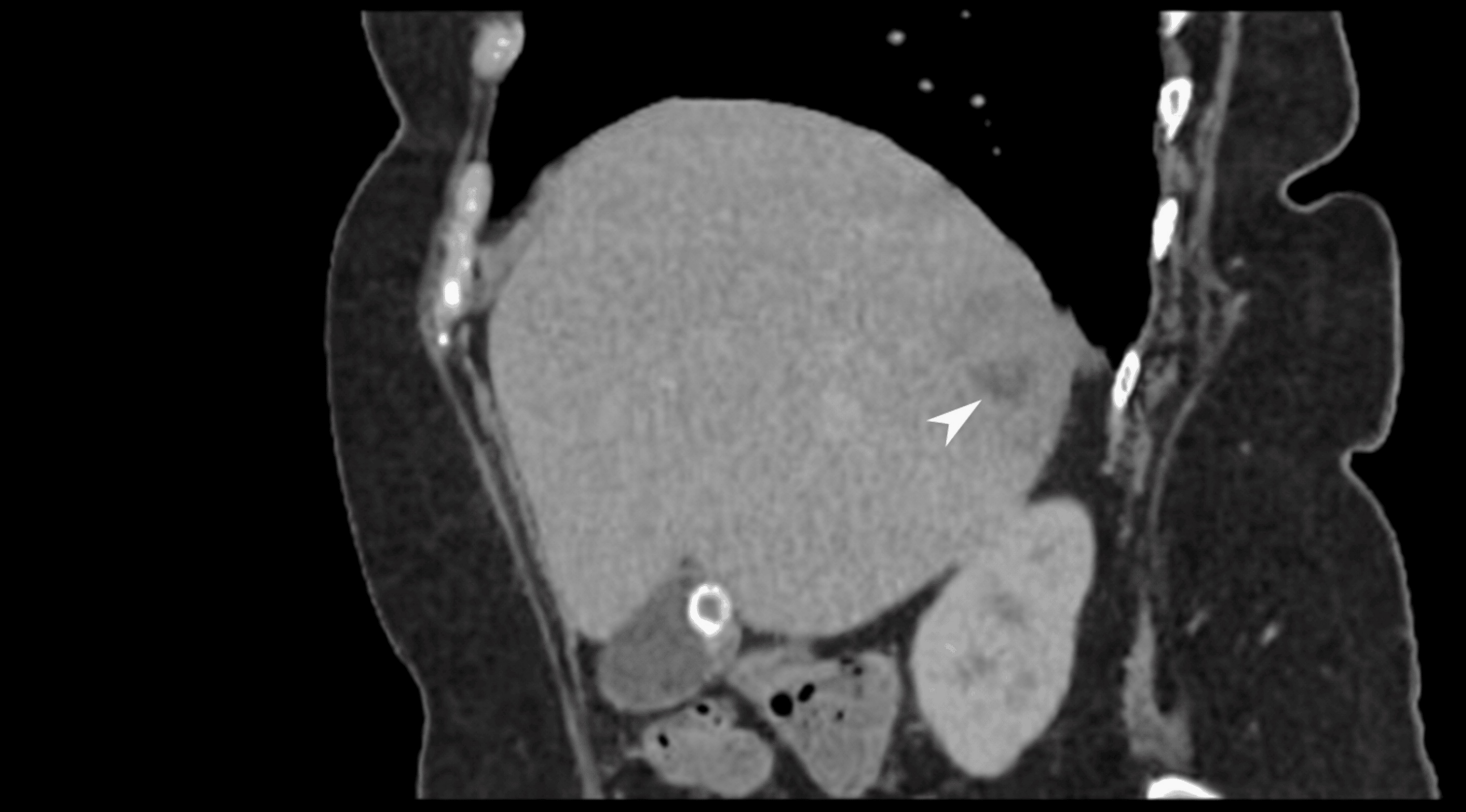
Coronal CT abdomen White arrowhead highlighting a hypodense lesion within the hepatic parenchyma, demonstrating subtle peripheral enhancement, consistent with a resolving hepatic abscess. The appearance reflects a residual intrahepatic fluid collection in the context of interval improvement; CT: Computerized tomography.

**Figure 4:**
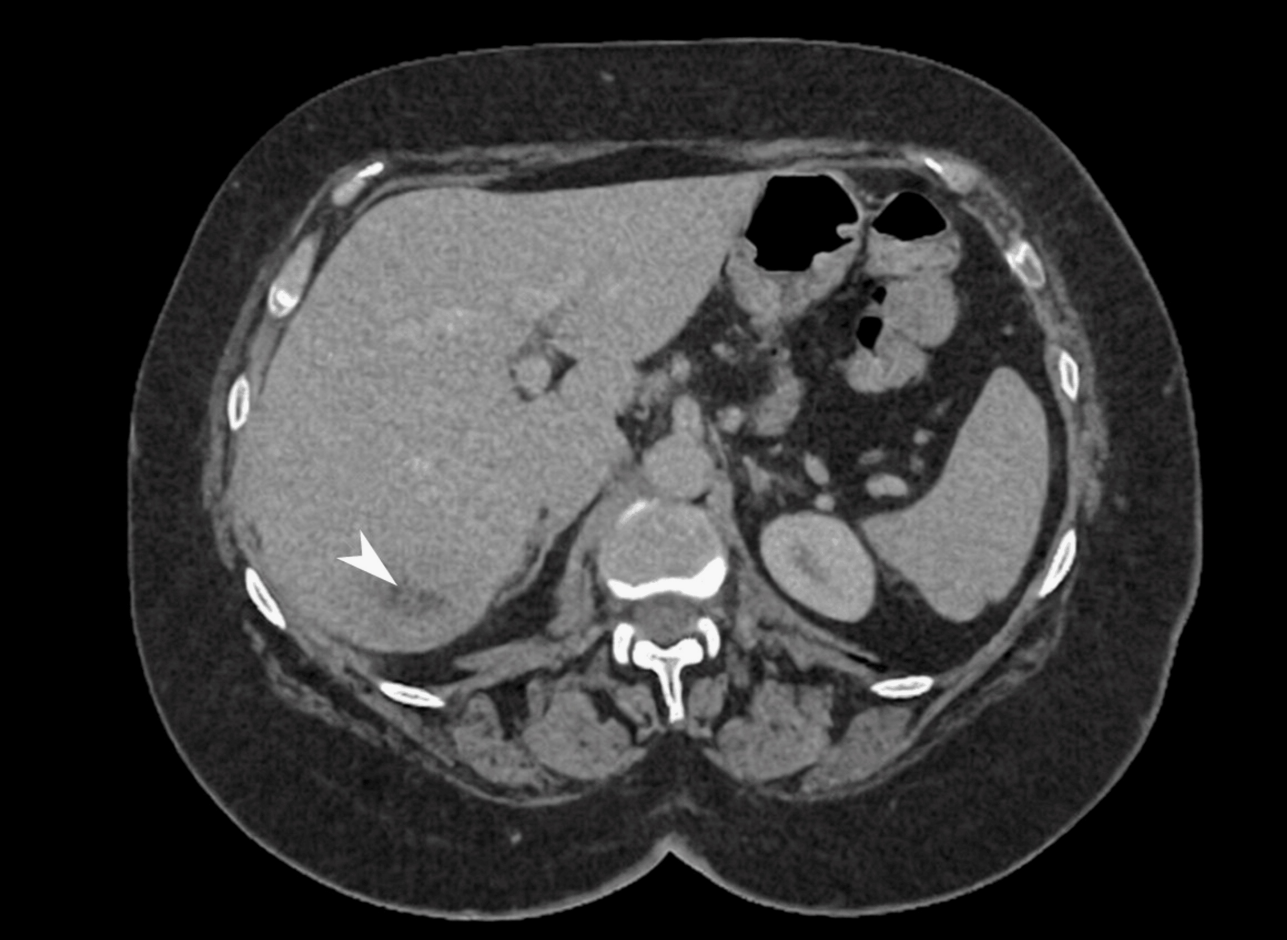
Repeated sagittal image of CT abdomen White arrowhead indicates a focal low-attenuation lesion in the right hepatic lobe with mild peripheral enhancement, consistent with a resolving hepatic abscess. The reduced conspicuity and absence of aggressive features support interval improvement of the infective collection; CT: Computed tomography.

The Interventional Radiology team assessed the patient and although the largest abscess measured 9.5 × 8.3 × 7.2 cm, conservative management was advised due to clinical improvement and broad-spectrum antibiotics were continued. By day 11, clinical improvement was noted, as she was no longer febrile and inflammatory markers showed a declining trend. Hemoglobin dropped to 89 g/L (reference range: 115-160 g/L) without overt bleeding. Colonoscopy was planned to investigate iron deficiency anemia and scheduled after treatment of the acute phase. A repeat CT scan two weeks later showed interval reduction of abscess size, but with a new ascending colon wall thickening (Figures 3, 4).

**Figure 5:**
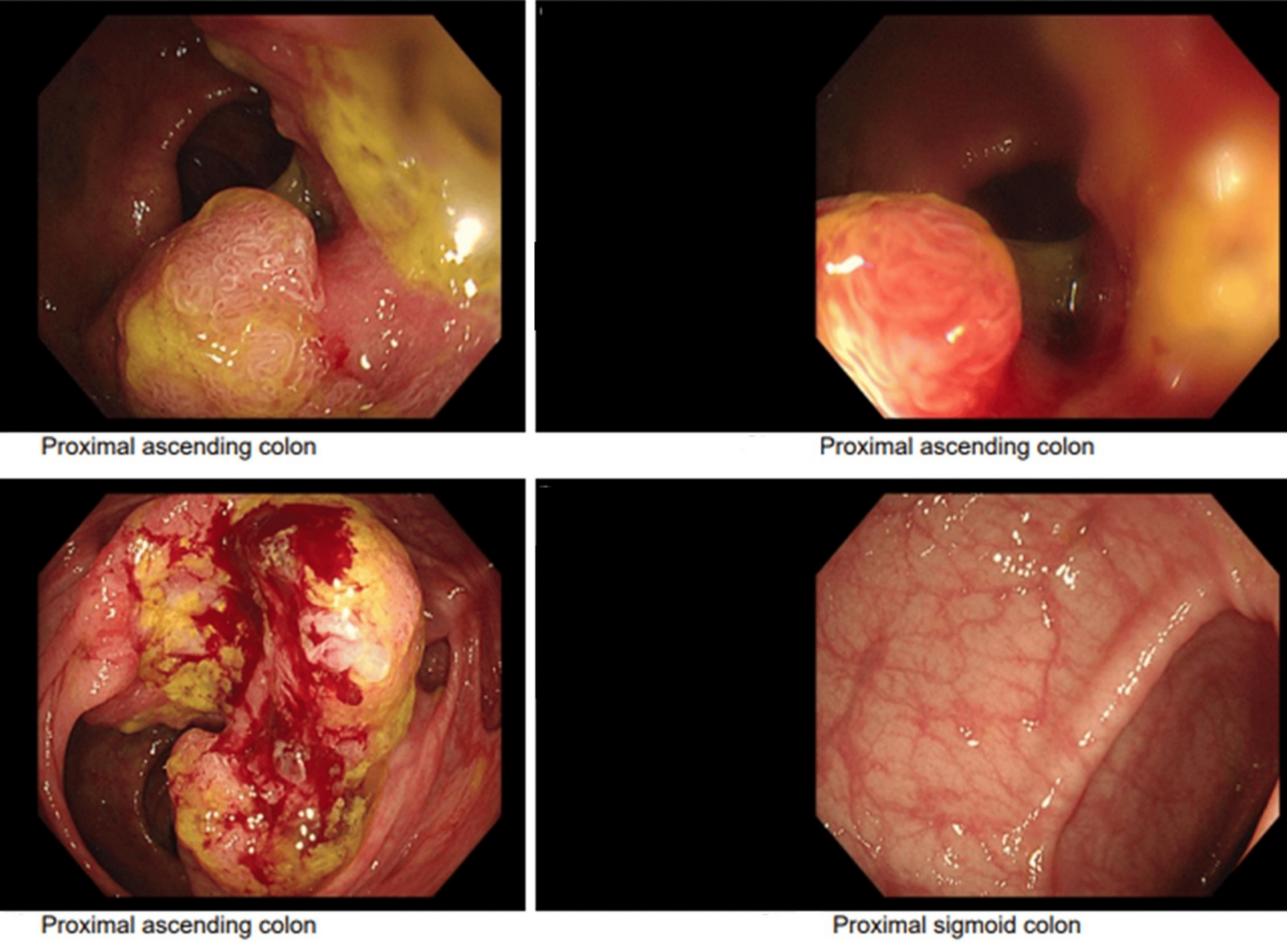
Colonoscopy images Showing the thickened lesion in the ascending colon which was described in the repeat CT scan; biopsies were sent for examination.

The patient was discharged on day 18 with oral antibiotics. Two weeks later, she presented again to the emergency department with abdominal pain, anemia, and diarrhea. Stool culture using rapid test kit confirmed *Clostridioides difficile*, treated with oral vancomycin. CT imaging again demonstrated persistent but improving abscesses and ascending colon thickening. Colonoscopy revealed a suspicious lesion in the ascending colon, however biopsy of the same showed adenoma with moderately differentiated cells (Figure 5).

**Figure 6:**
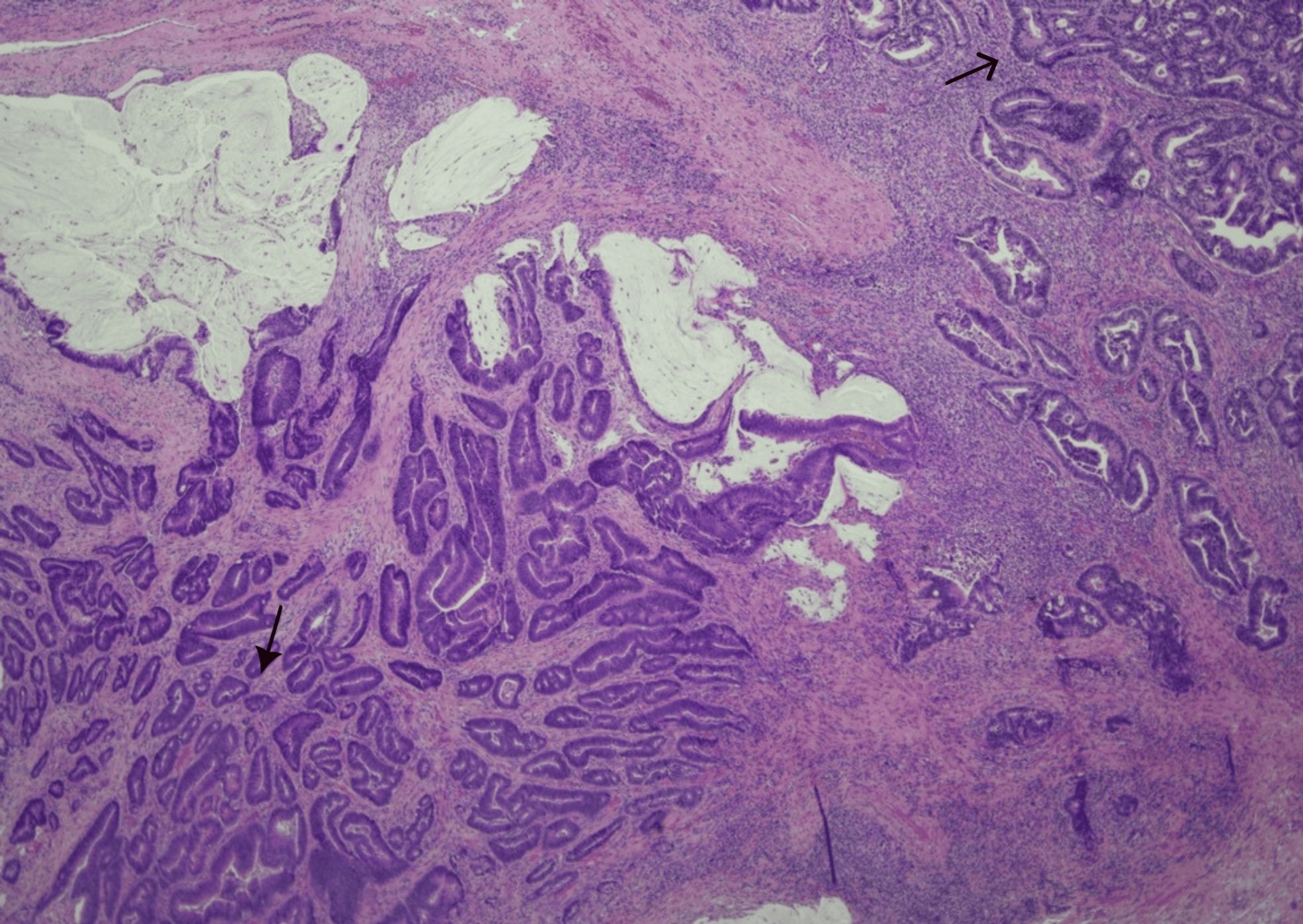
Histological section of colorectal adenocarcinoma Showing invasive tumor glands with associated mucin pools and stromal reaction. Neoplastic glands infiltrating the surrounding tissue with moderate glandular differentiation observed. Image is 10 times magnified with Hematoxylin–eosin (H&E) staining. Dark arrow indicates irregular neoplastic glands infiltrating the surrounding tissue; Slim arrowhead indicates desmoplastic stromal reaction pattern.

**Figure 7:**
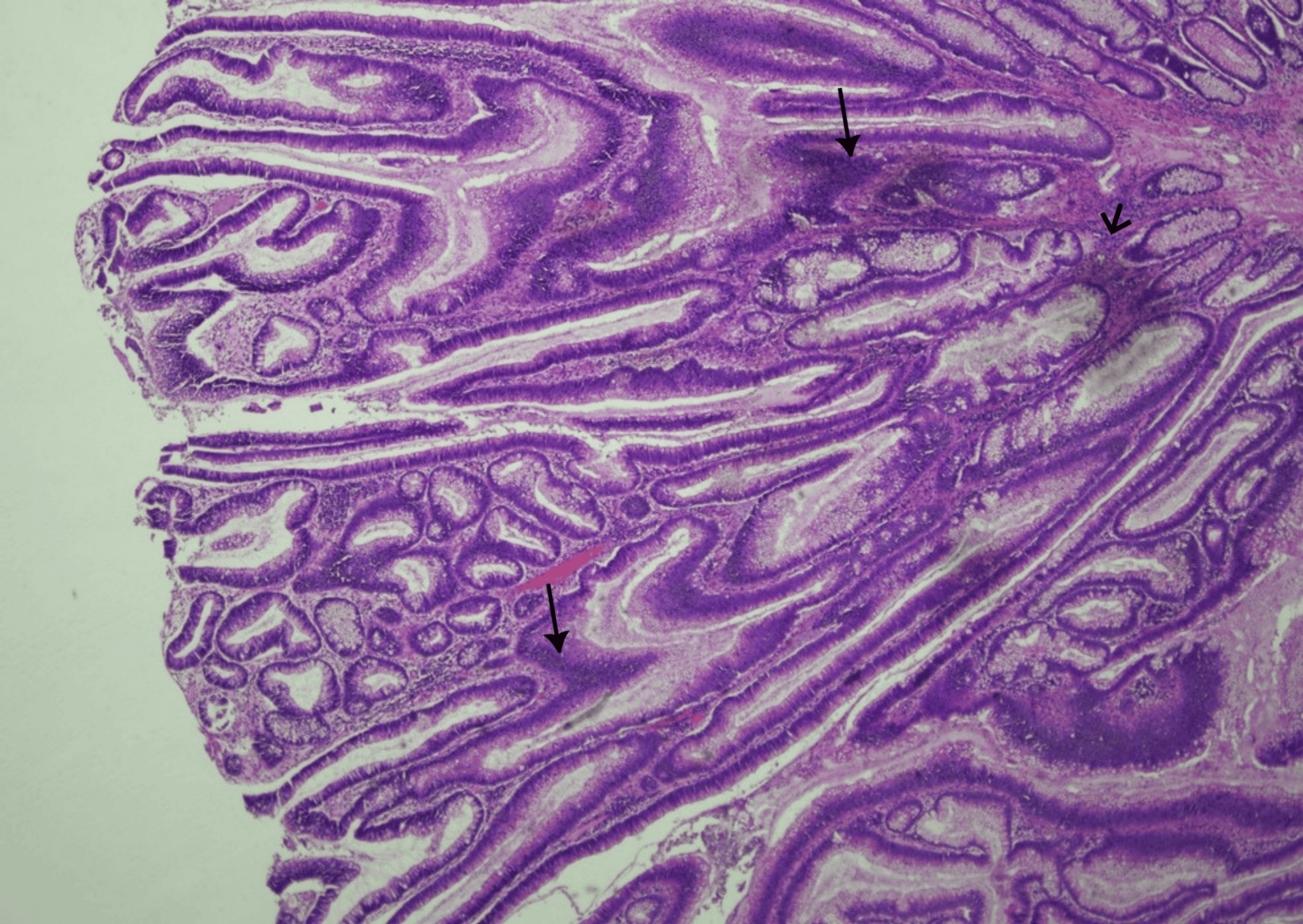
Neoplastic glands show complex branching and infiltration within the surrounding stroma Image is 10 times magnified with Hematoxylin–eosin (H&E) staining. Dark arrow indicates irregular neoplastic glands infiltrating the surrounding tissue; Slim arrowhead indicates desmoplastic stromal reaction pattern.

Her case was discussed in the surgical multidisciplinary team meeting, and she was planned for a right hemicolectomy two months later. Histopathology of the resected bowel showed a moderately differentiated adenocarcinoma (stage II) without nodal metastasis (Figures 6, 7).

Post-surgical recovery was uneventful. The patient was referred to the oncology multidisciplinary team for further management.

## Discussion

This case highlights several diagnostically challenging and clinically relevant aspects of PLA. The patient’s presentation was typical for PLA, yet the absence of an obvious hepatobiliary source, negative tumor markers, and persistent inflammatory response required a broader diagnostic consideration, ultimately leading to the discovery of a cryptic colon primary [2,16,17]. PLA remains relatively uncommon in high-income regions, but its clinical importance lies in its role as a potential sentinel event for occult malignancy rather than in its absolute incidence [1,9-11].

Population-based studies from Taiwan, Korea, and Western cohorts demonstrate a three- to seven-fold increased, time-dependent risk of CRC following pyogenic liver abscess, most pronounced within the first six months and largely confined to patients without an identifiable biliary source, supporting a targeted strategy for early lower GI evaluation in cryptogenic cases [9-11]. This relationship is believed to arise from the disruption of the colonic mucosal barrier by an underlying neoplasm, permitting portal venous bacterial translocation and hepatic seeding, rather than from any organism-specific effect [12,13].

This patient’s unexplained iron deficiency anemia and colonic wall thickening were key clues pointing toward this underlying malignancy. The eventual diagnosis of ascending colon adenocarcinoma emphasizes the importance of thorough lower gastrointestinal evaluation in cryptogenic PLA, as consistently recommended in the literature [9-11,14,15]. In our case, with CRC diagnosed within months of the PLA, the timing aligns perfectly with the high-risk window identified by cohort studies [10,11] and mirrors findings in other case reports in which PLA was the sentinel event [14,18].

Another noteworthy feature is the successful conservative management of large, multiseptated abscesses. While current guidelines typically recommend drainage for abscesses of size more than 5 cm due to concerns about antibiotic penetration and treatment failure [4], emerging evidence suggests that a subset of stable patients with improving inflammatory markers may respond well to antibiotics alone [16,17]. Our patient’s favorable outcome, despite an abscess nearly 10 cm in size, strengthens this evolving perspective and underscores the value of individualized, multidisciplinary assessment over rigid protocol-based care [14,17]. This approach can spare patients the potential risks of an invasive procedure, such as bleeding or secondary infection.

## Conclusions

Cryptogenic PLAs are strongly associated with underlying CRC and should prompt thorough lower GI evaluation, especially in patients with iron deficiency or abnormal imaging. Early recognition of PLA as a sentinel event allows timely diagnosis of occult malignancy. Furthermore, it demonstrates that conservative medical therapy may be a viable and effective strategy even for large abscesses in the context of careful clinical monitoring and a multidisciplinary team decision. Individualized, multidisciplinary assessment remains key to optimizing outcomes.
